# Bedaquiline- and clofazimine- selected *Mycobacterium tuberculosis* mutants: further insights on resistance driven largely by *Rv0678*

**DOI:** 10.1038/s41598-023-36955-y

**Published:** 2023-06-27

**Authors:** J. Snobre, M. C. Villellas, N. Coeck, W. Mulders, O. Tzfadia, B. C. de Jong, K. Andries, L. Rigouts

**Affiliations:** 1 Mycobacteriology Unit, Biomedical Sciences, Institute of Tropical Medicine, Antwerp, Belgium; 2 Internal Medicine Department, UZ Brussel, Brussels, Belgium; 3 Doctoral School of Life Sciences & Medicine, Vrije Universiteit Brussel, Brussels, Belgium; 4 Department of Infectious Diseases, Janssen Pharmaceutica, Beerse, Belgium

**Keywords:** Diseases, Microbiology, Bacteria, Infectious-disease diagnostics

## Abstract

Drug-resistant tuberculosis is a serious global health threat. Bedaquiline (BDQ) is a relatively new core drug, targeting the respiratory chain in *Mycobacterium tuberculosis* (Mtb). While mutations in the BDQ target gene, *atpE,* are rare in clinical isolates, mutations in the *Rv0678* gene, a transcriptional repressor regulating the efflux pump MmpS5-MmpL5*,* are increasingly observed, and have been linked to worse treatment outcomes. Nevertheless, underlying mechanisms of (cross)-resistance remain incompletely resolved. Our study aims to distinguish resistance associated variants from other polymorphisms, by assessing the in vitro onset of mutations under drug pressure, combined with their impact on minimum inhibitory concentrations (MICs) and on protein stability. For this purpose, isolates were exposed in vitro to sub-lethal concentrations of BDQ or clofazimine (CFZ). Selected colonies had BDQ- and CFZ-MICs determined on 7H10 and 7H11 agar. Sanger sequencing and additional Deeplex Myc-TB and whole genome sequencing (WGS) for a subset of isolates were used to search for mutations in *Rv0678, atpE* and *pepQ*. In silico characterization of relevant mutations was performed using computational tools. We found that colonies that grew on BDQ medium had mutations in *Rv0678*, *atpE* or *pepQ*, while CFZ-exposed isolates presented mutations in *Rv0678* and *pepQ*, but none in *atpE*. Twenty-eight *Rv0678* mutations had previously been described among in vitro selected mutants or in patients’ isolates, while 85 were new. Mutations were scattered across the *Rv0678* gene without apparent hotspot. While most *Rv0678* mutations led to an increased BDQ- and/or CFZ-MIC, only a part of them surpassed the critical concentration (69.1% for BDQ and 87.9% for CFZ). Among the mutations leading to elevated MICs for BDQ and CFZ, we report a synonymous Val1Val mutation in the *Rv0678* start codon. Finally*, *in silico characterization of *Rv0678* mutations suggests that especially the C46R mutant may render *Rv0678* less stable.

## Introduction

Emergence of drug-resistant TB (DR-TB) represents a major challenge for TB control programs. Treatment of DR-TB includes complex, multidrug regimens, with serious side effects and low cure rates^1^. The World Health Organization has responded to this challenge updating guidelines to treat multidrug-resistant (MDR-)TB to include new- and repurposed drugs, the most promising of which seems to be bedaquiline (BDQ), a diarylquinoline targeting subunit C of the ATP synthase in the respiratory chain, a novel mechanism in *Mycobacterium tuberculosis* (Mtb)^2–4^. According to the latest guidelines, shorter all-oral BDQ-including regimens can be used as an alternative to standardized regimens with injectable agents^5^. Also, a shorter regimen with BDQ, pretomanid and linezolid may be used to treat patients with extensively drug resistant (XDR-)TB as an alternative to the longer regimen^6,7^. With the wider implementation of BDQ, acquired resistance has been reported, linked in some cases to worse treatment outcomes^8,9^. While mutations in BDQ’s direct target gene (*atpE*) are rare^10–12^, most strains phenotypically resistant to BDQ show mutations in *Rv0678*^10,13^. This gene is thought to be responsible for regulation of the MmpL5-MmpS5 efflux pump^14,15^.There is evidence that mutations in *Rv0678* concurrently lead to resistance to CFZ, a repurposed drug also targeting the respiratory chain^15^, although the level of this cross-resistance is poorly understood. A key knowledge gap consists of the correlation between specific mutations and their effect on phenotypic resistance and treatment outcome, especially when identified at baseline in patients not previously exposed to BDQ or CFZ. For most other TB drugs, resistance conferring mutations concern a limited number of codons^16^. In contrast, *Rv0678* presents a wide range of mutations with variable effect on MICs, and not all resulting from prior known BDQ or CFZ drug exposure^13^. Closing this gap in understanding the correlation between mutations and phenotypic BDQ resistance is crucial for correct interpretation and development of molecular assays, treatment choice, and thus for prevention of further emerging resistance to BDQ before its activity is lost.

Previous in vitro approaches have tried to close this genotypic-phenotypic gap^13,17–21^. This work seeks to complement those studies, by describing additional mutations linked to BDQ and CFZ resistance in Mtb following in vitro drug exposure, focusing on *Rv0678, atpE* and *pepQ* genes. In addition to their phenotypic (MIC) impact, we characterize the effect of nonsynonymous single nucleotide polymorphisms (SNPs) on protein stability and 3D secondary structure using a computational approach.

## Materials and methods

### In vitro selection of resistant Mtb mutants

This study comprises data from three separate in vitro selection efforts. For Set I BDQ selection had been previously done at the Swedish Institute for Infectious Disease Control, Solna, Sweden^22^. For sets II BDQ selection was done at Janssen Pharmaceutica, Beerse, Belgium, and for Set III CFZ selection was done at the Institute of Tropical Medicine, Antwerp, Belgium (Table 1). Set I used only clinical Mtb isolates, while set II selected from two laboratory reference strains (H37Rv and CDC1551) and set III included both clinical- and laboratory derived mother strains. For set I and II the following approach was used. For each strain, a culture was grown to early stationary phase (OD_620_ 0.9–1) and adjusted to OD_620_ = 0.8 with Middlebrook 7H9 broth supplemented with 10% OADC and 0.05% Tween80. Two different volumes (100 µl and 1 ml) were plated on Middlebrook 7H10 agar (with 10% OADC) containing BDQ, at two different concentrations (0.3 µg/ml and 0.9 µg/ml), with 5 plates per condition. Plates were incubated for 4–6 weeks at 37 °C and colonies were counted and selected for subsequent MIC testing, storage and sequencing. For set III the approach differed in the fact that only 3 CFZ-containing plates were used and selected colonies were subcultures on Löwenstein Jensen slants. In addition, some of the selected colonies from Set III were further exposed to CFZ on 7H10 plates.

**Table 1:** Overview of in vitro selection methods and selected isolates 7H9-OADC-Tw80 = Middlebrook 7H9 broth supplemented with 10% OADC and 0.05% Tween 80; *7H10-OADC* Middlebrook 7H10 agar medium supplemented with 10% OADC, *Mtb* Mycobacterium tuberculosis, *LJ* Lowenstein-Jensen, *MDR* multidrug-resistant, *Lx* unknown lineage, *BDQ* bedaquiline, *CFZ* clofazimine, *MIC* minimal inhibitory concentration.

	Set I	Set II	Set III	
In vitro selection procedure				
Laboratory	SSI, Stockholm, Sweden	Janssen Pharmaceutica, Beerse, Belgium	ITM, Antwerp, Belgium	
Pre-culture medium	7H9-OADC-Tw80	7H9-OADC-Tw80	7H9-OADC-Tw80	
Selection medium	7H10-OADC	7H10-OADC	7H10-OADC	
Drug used	BDQ	BDQ	CFZ	
Drug concentration range	0.3–0.9 µg/ml	0.3–0.9 µg/ml	0.5–8 µg/ml	
Number of plates per condition	5	5	3	
Subcultured on	Plain 7H10 medium	Plain 7H10 medium	Plain LJ medium	
Subsequent selection on stepwise higher concentration	No	No	Yes	
Mtb mother strains used	Clinical isolates: P1 (L2, MDR) P3 (LX, MDR) P6 (L2, pan-susceptible)	Reference strains: H37Rv (L4, pan-susceptible) CDC1551 (L4, pan-susceptible)	Reference strain: H37Rv (L4, pan-susceptible) Clinical isolates: 02-3046 (L1, pan-susceptible) 11–1615 (L1, pan-susceptible) 01-1735 (L2, pan-susceptible) 01-1738 (L2, pan-susceptible) 02-1308 (L3, pan-susceptible) 02-1071 (L3, pan-susceptible) 10-1793 (L4, pan-susceptible) 10-1807 (L4, pan-susceptible)	

Results				Total
Total nr of selected colonies	85	80	103	268
Total nr of *Rv0678* and *atpE* sequenced	83	79	101	263
Total nr of failing *Rv0678* & successful *atpE* seq	2	1	2	5
nr of *Rv0678* mutants & *atpE* WT	71	73	94	238
intergenic mutation alone	3	3	3	9
intergenic & *Rv0678* mutation	0	0	1	1
*atpE* mutants	5	3	0	8
*pepQ* mutants per mutants tested	4 on 6	0	0 on 21	4
nr of *Rv0678* WT & *atpE* WT & *pepQ* WT	0	0	3	3
Nr of MIC-CFZ/BDQ for successfully sequenced isolates	81	63	78	222

### Minimal inhibitory concentration (MIC) determination

The MIC for BDQ was determined on Middlebrook 7H11 agar medium at a concentration range of 0.008 to 2 µg/ml as described before^23^, while CFZ was tested on 7H10 agar at a range of 0.008 to 8 µg/ml, with three or four weeks of incubation. The H37Rv Mtb reference strain was included as a control for each batch of medium and presented an MIC of 0.0625 µg/ml for BDQ and 0.5 µg/ml for CFZ (+/− one dilution).

### Sequencing of *Rv0678* and *atpE* genes

All isolates had Sanger sequencing done for *Rv0678* and *atpE*, while only those showing a wildtype (WT) sequence for both genes, or a synonymous *Rv0678* mutation had *pepQ* sequenced in addition. To this end, a DNA fragment containing *Rv0678* and part of the intergenic region between *mmpS5* and *Rv0678* was amplified by PCR using primers described in Table S4. Primers for *atpE* and *pepQ* are also described in Table S4. Boiled cultures, prepared by transferring a loopful of freshly grown bacilli in 400 µl Tris–EDTA buffer (10 mM Tris, 1 mM EDTA, pH 8.0) and heating for 5 min at 100 °C, were used as DNA template for PCR. The PCR products were sequenced at BaseClear (The Netherlands), using the respective primers. For sequence analysis, CLC Workbench software was used with H37Rv as reference (NC_0009623)^24^. Additional Deeplex Myc/TB (Genoscreen, France) and whole genome sequencing (WGS) analysis was performed on isolates presenting a WT *Rv0678, atpE* and *pepQ* gene. Deeplex Myc/TB (Genoscreen, Lille, France) was run on boiled cultures described above following the kit’s instructions. For WGS we followed the procedure described in previous publications^25^. Briefly, genomic DNA (gDNA) extraction was performed on growth from fresh Löwenstein-Jensen slants and after an in-house developed lysis protocol^26^, the semi-automated Maxwell 16 Cell DNA kit was used to purify the extracted gDNA according to the manufacturer’s instructions. Extracted gDNA was sequenced on an Illumina MiSeq platform using the Illumina Nextera XT DNA Library preparation Kit.

### *Rv0678*,* atpE*, *pepQ* mutants literature search

Detected mutations were compared to those in the public literature described in clinical isolates and previous in vitro studies through November 2022. Only studies using WHO approved phenotypic drug-susceptibility testing methods were included. Mutations were accompanied, when available, with BDQ- and CFZ-MIC values and information about previous drug exposure.

### Free energy calculation with FoldX

We performed a free energy calculation for point mutations in the available protein structures of *Rv0678, atpE* and *pepQ* using FoldX 5^27^ to predict the change in protein stability they may cause. Frameshifts and mutations affecting the promoter region were excluded from the analysis, as these are not supported by FoldX. The stability change in FoldX, ΔΔ*G* (kcal/mol), was computed as the difference between the average stability of mutant and WT protein structures. When the ΔΔ*G*-value was > 0, a mutation was considered destabilizing, while with a ΔΔ*G*-value < 0 the mutation was classified as stabilizing. The error margin of FoldX is approximately 0.5 kcal/mol, so changes in that range (either positive or negative) were not considered as significant.

### Protein structure visualization with Alpha Fold

Protein structures were visualized with Alpha Fold^28^, a computational tool that predicts protein structures with an accuracy comparable to experimental structures^28^. WT amino acid sequences for *atpE*, *Rv0678* and *pepQ* were obtained from Mycobrowser, a genomic and proteomic data repository for pathogenic mycobacteria^29^. Next, ColabFold^30^ was run locally to predict the mutant protein structure, starting from the mutated sequence. Cartoon diagram of predicted three-dimensional structure was generated by YASARA.

### Promoter prediction analysis

To investigate the role of mutations in the *Rv0678* promoter region, we used BPROM software^31^, a bacterial sigma70 promoter recognition program and NNPP Promoter Prediction^32^, that uses Neural Networks to detect transcription start sites. Analysis was run on the 350 bp upstream region obtained from Mycobrowser^29^.

## Results

### Genotypic characterization of BDQ- and CFZ-selected isolates from this study

Two-hundred sixty-eight colonies were selected from agar plates supplemented with BDQ or CFZ, of which 263 had successful *Rv0678* and *atpE* Sanger sequences, while for five no *Rv0678* amplicon could be obtained using our primers (Table 1; detailed information on all mutations is provided in Table S1). The majority (238/263; 90.5%) of selected isolates harbored a mutation only in *Rv0678*, regardless of the selecting drug (Table 1). None of the CFZ-selected isolates carried an *atpE* mutation, while only 8/162 (4.9%) isolates subjected to BDQ pressure had a mutated *atpE* gene. *PepQ* sequencing was performed for a total of 28 isolates with either WT *Rv0678* and *atpE* sequences (n = 15), those with failing *Rv0678* and having WT *atpE* (n = 5), or having a synonymous *Rv0678*_Val1Val mutation and a WT *atpE* sequence (n = 8). Mutations in *pepQ* were observed in four of 28 isolates tested, all of them selected under BDQ pressure. For the 11 isolates presenting a WT profile for all three genes by Sanger sequencing, 10 had additional Deeplex (7 isolates) or WGS analysis (3 isolates) testing done, of which eight revealed an *Rv0678* mutation, five of them showing minority variants (from 1 to 84.25%).

**Figure 1:**
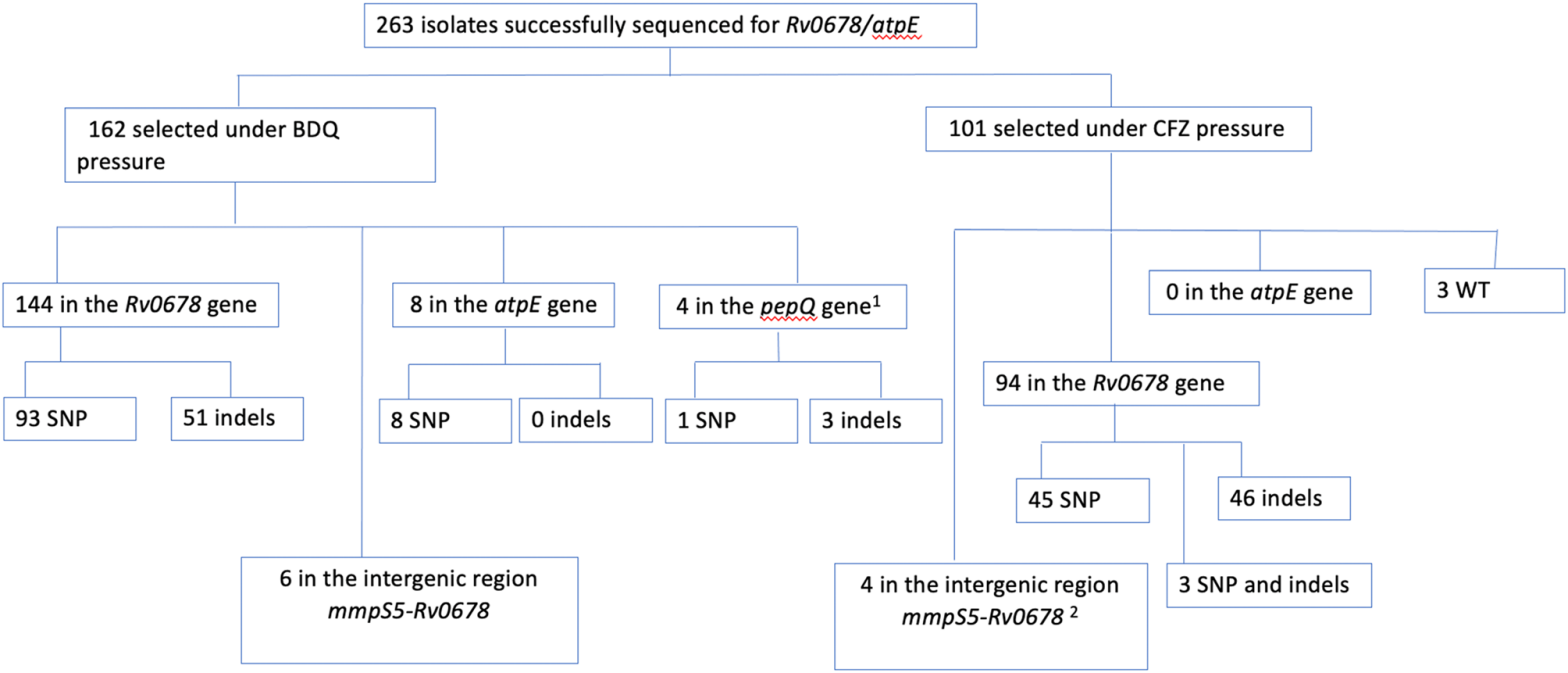
Schematic visualization of in vitro selected mutants stratified per selecting drug. ^1^PepQ gene was sequenced only in a subset of 28 isolates. ^2^One of these isolates presented a mutation both in the intergenic region and in Rv0678.

Overall, none of the mutants harbored mutations in more than one of the genes tested. *Rv0678* mutations were spread over the entire gene with a high proportion of indels (42,1% versus 57.9% SNPs) (Fig. 1), and no single hotspot could be identified (Fig. 31 and 41). Six different intergenic mutations occurred, of which 4 SNPs and 2 insertions. One of these SNPs (− 30 A→G) was found in combination with a SNP in the *Rv0678* gene, three other SNPs occurred at nucleotide − 8, where “T” was replaced by “A”, “C” or “G”, while two insertions (Ins A) occurred at nucleotide − 9 and − 10. A synonymous Val1Val mutation conferring high BDQ (0.5 µg/mL and CFZ (2 µg/mL) MICs was detected in isolates selected under CFZ pressure, without acquisition of additional mutations in *Rv0678*, *atpE* or *pepQ*. Also, heteroresistant profiles detectable by Sanger sequencing (double peaks showing WT + mutant) were seen in 14 different CFZ-selected *Rv0678* mutants (29 isolates), and among 3 different BDQ-selected *Rv0678* mutants (3 isolates).

All *atpE* mutations were SNPs and detected at amino acid positions 28, 61, 66 and 63 (Fig. 61), while the 4 *pepQ* mutations were one SNP and 3 indels. Eighteen isolates presented multiple mutations in *Rv0678*. No double mutations or heteroresistance were observed in either *atpE* or *pepQ,* albeit most were tested by Sanger sequencing.

### Phenotypic susceptibility testing of in vitro BDQ- and CFZ-selected isolates from this study

MIC data were available for 222 of 263 successfully sequenced isolates, with the rest missing due to inadequate growth of the control (n = 2) or non-availability of the isolates after subculturing (n = 39).

**Figure 2:**
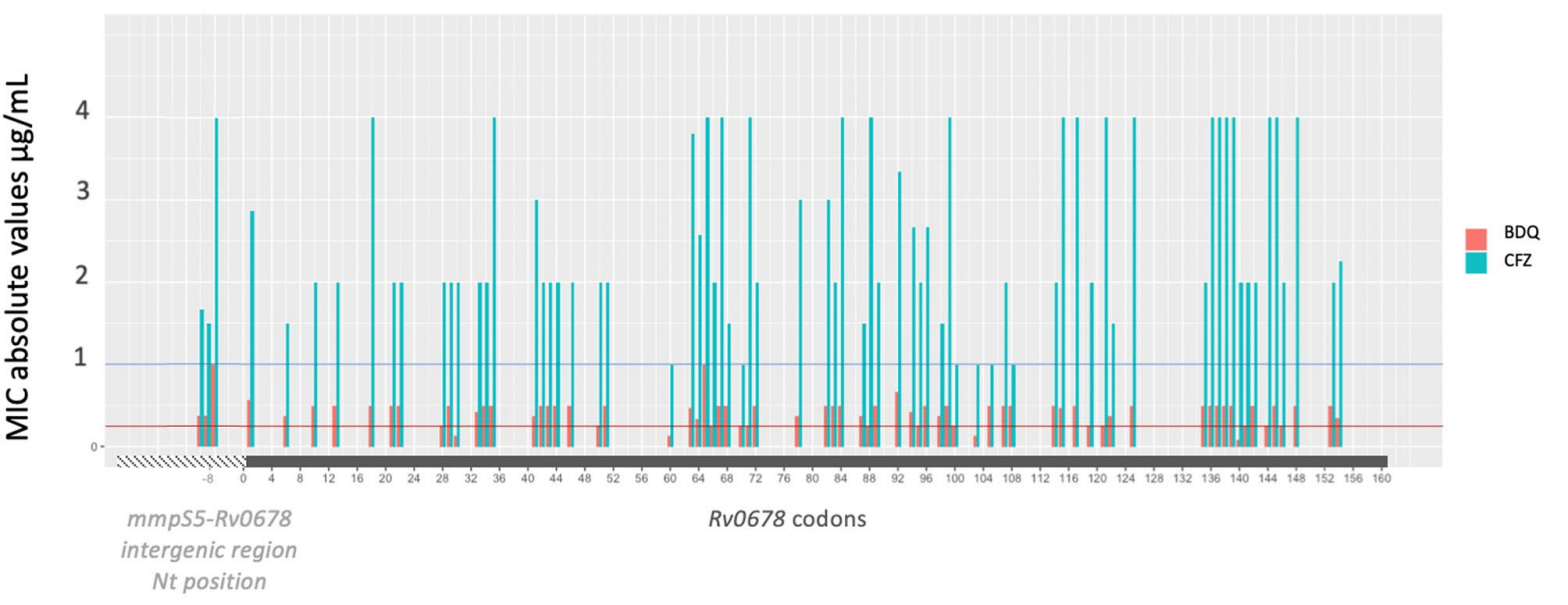
Mutations’ coding position and associated bedaquiline and clofazimine minimal inhibitory concentration mean values throughout Rv0678. Only mutations observed in this study are represented. Proposed critical concentrations for clofazimine (blue) and bedaquiline (red) are represented with a straight line at 1 µg/mL and 0.25 µg/mL respectively.

Applying the EUCAST and WHO recommended resistance cutoff of > 0.25 µg/mL and > 1 µg/mL, most *Rv0678* mutants presented an increased BDQ MIC (69.1%) and CFZ (87.9%) exceeding the respective critical concentrations of 0.25 µg/mL and 1 µg/mL with 63.5% of the isolates exceeding both critical concentrations. (Fig. 2). Six (66.7%) of 9 isolates carrying a mutation in the intergenic region and available for testing presented a phenotypically resistant BDQ- and CFZ-MIC. Mutations in the *atpE* gene presented BDQ phenotypic resistance in 5/7 isolates available for testing. The two BDQ-susceptible *atpE* mutants had an MIC at the cut-off (one each with Glu61Asp and Asp28Gly). The two Ala63Pro mutants were also found CFZ resistant, while the other *atpE* mutants were CFZ susceptible. All the isolates carrying a *pepQ* mutation showed phenotypic CFZ resistance, while none of them presented a BDQ MIC above the critical concentration.

Next, we measured the fold increase in MICs for BDQ and CFZ compared to the respective ancestor (“mothers”). For a subset of isolates selected under CFZ pressure and with available MIC (30/78) it was not possible to calculate the fold increase as some mother strains were no longer available for paired MIC testing.

**Figure 3:**
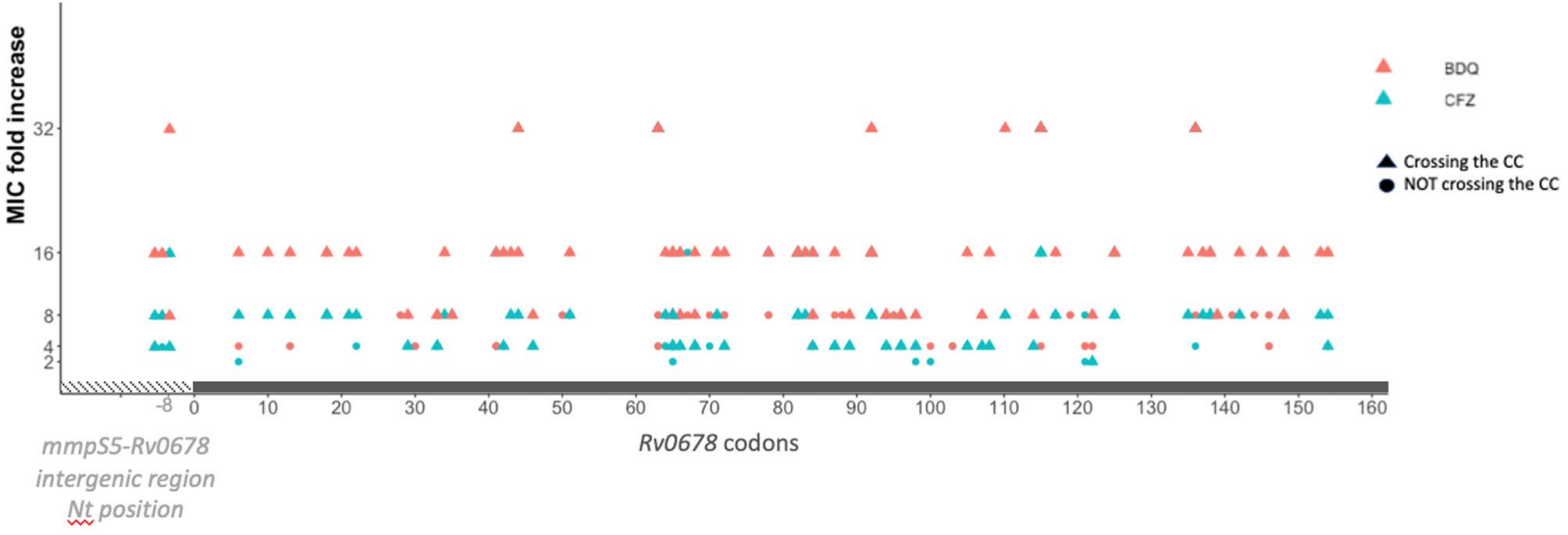
Fold increase in minimal inhibitory concentrations (MIC) for bedaquiline and clofazimine between baseline isolates and the respective selected spring off after in vitro drug exposure. No clear association was found between affected codons in the spring off and bedaquiline (BDQ, red) or clofazimine (CFZ, blue) MIC fold increase. When the MIC of the in vitro selected isolates surpassed the drug’s critical concentration this is indicated with triangles. *CC* critical concentration; *Nt* nucleotide.

**Figure 4:**
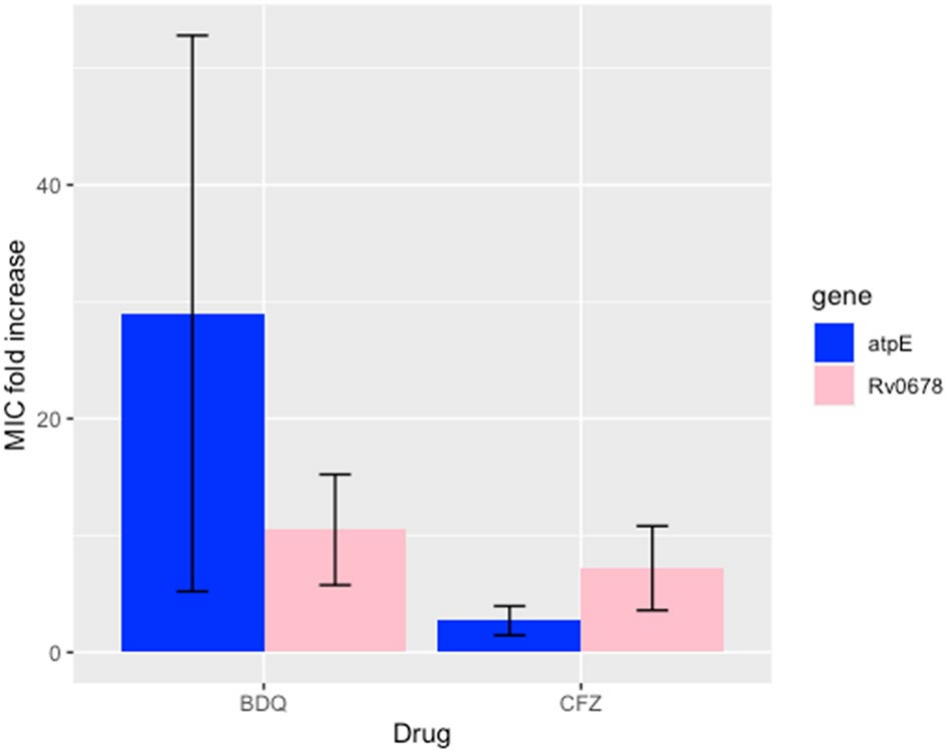
Fold increase in minimal inhibitory concentration (MIC) for bedaquiline (BDQ) and clofazimine (CFZ) in isolates selected in vitro compared to the wild type mother strain, stratified per selecting drug (BDQ or CFZ) and the affected genes (atpE (blue) or Rv0678 (pink)).

All isolates that acquired a mutation in *Rv0678* presented a BDQ and CFZ MIC fold increase ranging from four to 32 (Fig. 41). Specifically, half of *Rv0678* mutants (53.1%; 92/173) presented a high BDQ MIC increase of 16–32 fold, while this was moderate (four–eightfold) for the remaining half (46.8%; 81/173). As for CFZ, only one third (27.2%; 47/173) of *Rv0678* mutants presented a high MIC fold increase of 16–32 and majority (74.5%; 126/173) a moderate to low increase of 2–8. No clear association between affected *Rv0678* codons and MIC fold increase could be found (Fig. 3). The fold increase in MIC was not associated with the selecting drug. Isolates carrying a mutation in *atpE* presented a higher BDQ MIC fold increase with 2/7 isolates presenting a 64 fold increase, another 2/7 isolates an MIC of 16–32 fold and the remaining 3/7 an MIC increase of eightfold. In contrast, isolates with a mutation in *atpE* presented lower CFZ MIC (one–fourfold) increase (Fig. 4).

### Comparing mutations occurring among clinical versus in vitro selected isolates

**Figure 5:**
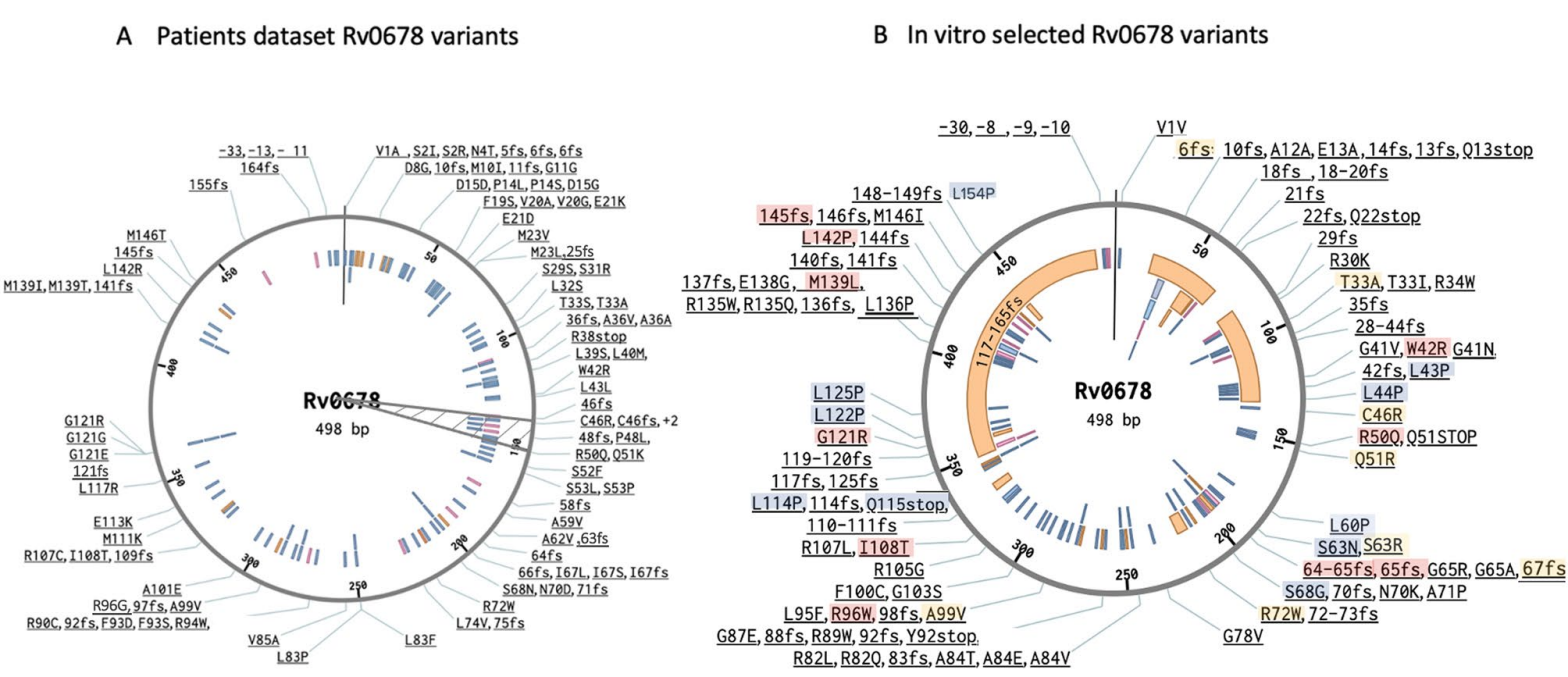
Overview of published patient derived- (**A**) compared to in vitro selected Rv0678 mutations from our study (**B**). Mutations in the intergenic region are included. At the inner circle, deletions are depicted in orange, insertions in pink and SNPs in blue. (**A**) The grey triangle highlights the region reported to show the most frequent Rv0678 mutations observed in bedaquiline-resistant patients in South Africa^9^. (**B**) Mutations described in this study are highlighted in red if already reported in patient isolates, in blue if already reported in other in vitro studies, and in orange if previously reported in both clinical and in vitro selected isolates.

For this purpose, we searched in published literature^8,9,15,17,20,33,34,34,35^ through November 2022. In total, we found 202 different mutations from clinical isolates with elevated MICs reported for BDQ and/or CFZ: 180 in *Rv0678*, 12 in *atpE*, three in the intergenic region between *mmpS5* and *Rv0678,* and *7* in the *pepQ* gene (Table S2). Twenty of these clinical isolate mutations were shared with our in vitro dataset, of which 18 in the *Rv0678* gene (nt16delG, T33A, W42R, C46R, R50Q, Q51R, nt191-192insG, nt192-193insG, S63R, 67 fs, R72W, R96W, A99V, I108T, G121R, M139I, L142P, nt435delT) (Fig. 5). *Rv0678* mutations appeared both in BDQ-exposed (nt16delG, nt192insG, T33A, C46R, R96W, A99V, nt435delT) and -naïve patients (W42R, nt198insG), while for 8 mutations exposure information was not available. Although sometimes with different allele substitutions, all codons associated with in vitro selected resistance in *atpE* from this study were also found among published clinical isolates (codons 63, 61, 66)^13,36–38^.

**Figure 6:**
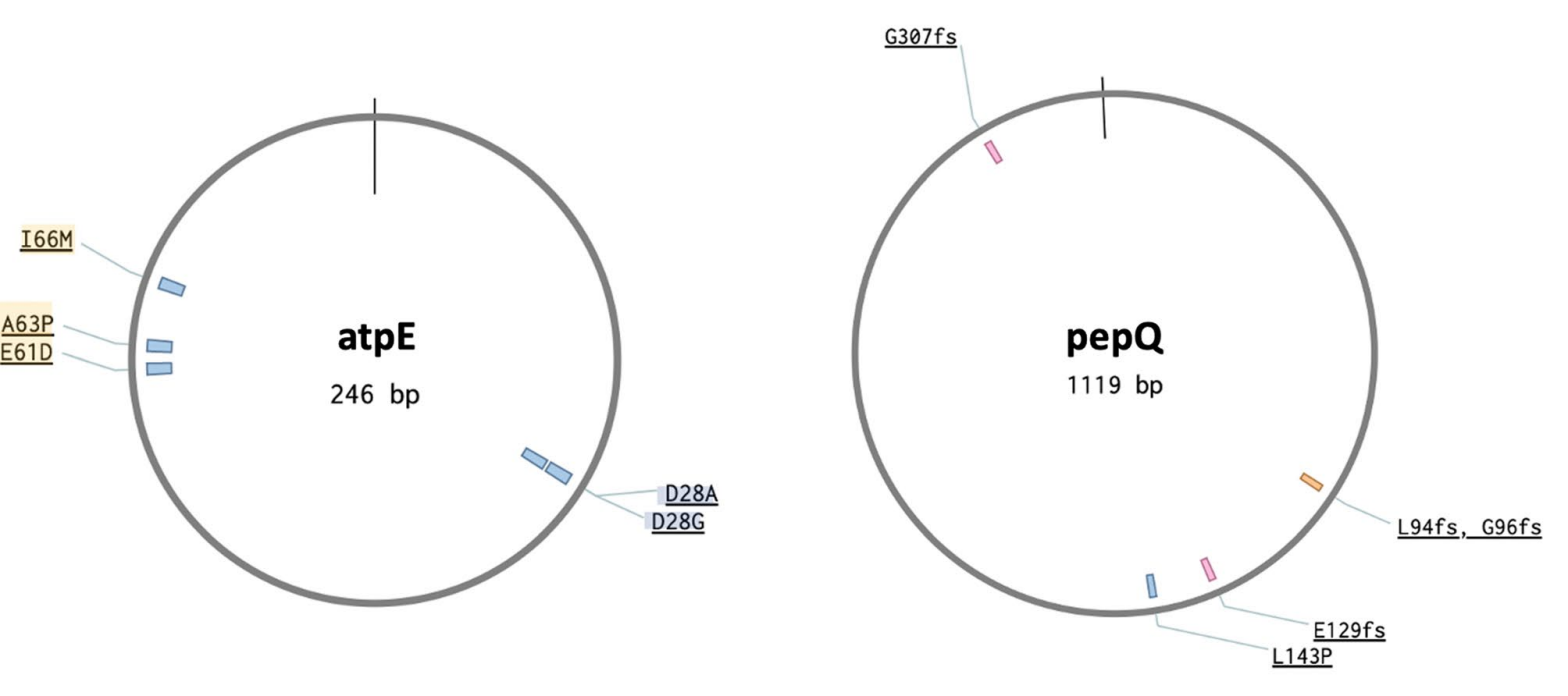
Overview of patients derived and in vitro selected atpE (Rv1305) and pepQ (Rv2535c) single nucleotide polymorphisms from our study and published datasets. At the inner circle, deletions are depicted in orange, insertions in pink and SNPs in blue. Mutations described in this study are highlighted in blue if already reported in other in vitro studies, and in orange if previously reported in both clinical and in vitro selected isolates.

From previous in vitro studies we extracted a dataset of 151 mutations associated with BDQ/CFZ resistance: 135 mutations in the *Rv0678* gene, 13 in *atpE,* and 3 in the *pepQ* gene (Table S3). Compared to previously published in vitro studies, 18 of our *Rv0678* mutations had already been described from lab selection alone (L43P, L44P, L60P, S63N, S68G, L114P, Q115*, L122P, L125P, L154P) or both in the lab and in patients (nt16delG, T33A, C46R, Q51R, S63R, 67 fs, R72W, A99V), leaving 85 as newly described *Rv0678* mutants (Fig. 5). To the best of our knowledge, none of the *pepQ* mutations had previously been described in vitro or in patients’ datasets (Fig. 6).

### Impact of observed *Rv0678*, *atpE* and *pepQ* mutations on protein stability

We investigated the effect of all nonsynonymous SNPs in *Rv0678*, *atpE* and *pepQ* reported in this study on protein stability using FoldX 5^27^ (Table S5).

**Figure 7:**
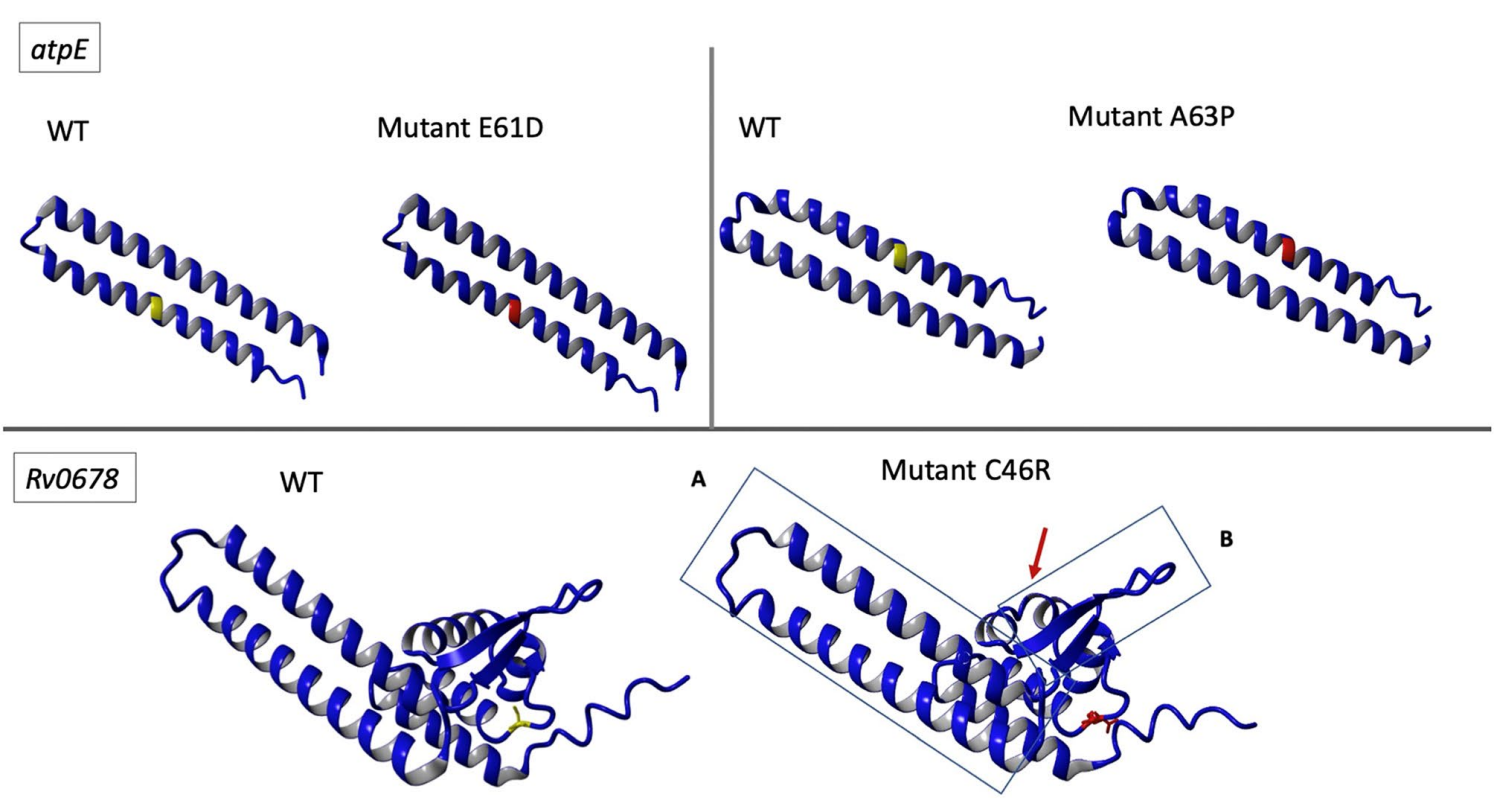
Secondary protein structure of atpE (E61D and A63P) and Rv0678 (C46R) mutants modelled with AlphaFold. Superimposition of wild type (WT) and mutated amino acids are shown respectively in yellow and in red. In the Rv0678 C46R mutant structure, dimerization (**A**) and DNA binding domain (**B**) are indicated. Changes in protein structure compared to the respective WT protein are highlighted with a red arrow.

The *Rv0678* folding stability calculation suggests that 44 out of 62 studied nonsynonymous SNPs associated with a BDQ- and CFZ-resistant phenotype have an impact on *Rv0678* protein folding/stability (ΔΔ*G* of > 0.500 kcal/mol) (Table S5). Mutant C46R showed the highest destabilizing effect with a ΔΔ*G* of > 10 kcal/mol. The affected structure of C46R (predicted with AlphaFold), shows changes in the DNA binding domain (Fig. 7). Although this mutant presented a phenotypic resistant profile, the observed MIC values were still moderate (BDQ MIC of 0.5 µg/mL, CFZ MIC of 2 µg/mL), and it caused not the highest MIC fold increase in our study. Overall, no significant correlation could be found between ΔΔ*G* and BDQ/CFZ MIC or MIC fold increase for *Rv0678* mutants (data not shown).

The *atpE* A63P and E61D mutations showed a predicted low grade destabilizing effect on protein stability with a ΔΔ*G* of 1.50 and 1.01 kcal/mol respectively (Fig. 7). Both mutations seem to be located in the BDQ binding domain, and already have been observed in clinical isolates. Only one nonsynonymous SNPs was available for *pepQ* (the other mutations being indels), leading to a ΔΔ*G* of 7.17 kcal/mol.

## Discussion

In this work we combined in vitro and computational approaches to measure the impact of isogenically selected *Rv0678*, *atpE* and *pepQ* mutations on BDQ and CFZ resistance in Mtb. Our findings result in a comprehensive dataset of 85 not previously reported mutations in the *Rv0678* gene, which all resulted in phenotypic BDQ/CFZ increase in MIC, albeit only 69.1% for BDQ and 87.9% for CFZ above the CC. We also describe a synonymous Val1Val mutation in the *Rv0678* gene conferring high BDQ and CFZ MICs. As expected, we did not identify *atpE* mutant selection with CFZ, although CFZ MICs were modestly increased in *atpE* mutants. Finally*,* we show that especially the C46R mutant, located in the *Rv0678* DNA binding domain, has an important predicted destabilizing impact on the *Rv0678* protein. In summary, we provide a broad catalogue of mutations associated to increased MICs, contributing to the effort towards the development of genotypic testing for BDQ resistance in DR-TB patients.

Reliable genotypic drug-susceptibility testing requires knowledge about which mutations are unequivocally associated with phenotypic resistance and which ones are not. The latter include lineage wide polymorphisms. As the clinical breakpoint of BDQ resistance is yet to be established, any impact of mutants on the MIC may be relevant. Consistent with previous in vitro and clinical studies^10,17,36,39^, in this work we show that most BDQ- and CFZ-MIC elevations can be explained by a single mutation in the *Rv0678* while a minority is driven by mutations in the *atpE* gene. *AtpE* mutants seem to be more prevalent in the first in vitro resistance study performed with BDQ (28%)^22^. While a previous study using clinical isolates showed that *Rv0678* mutations could lead also to hypersusceptibility with lower MIC^13^, in our study all *Rv0678* mutations in isogenically selected strains consistently increased the MIC. While *Rv0678* mutations mainly present low-medium BDQ increase in MIC, *atpE* mutations induced high-level BDQ-MIC fold increases, and some *Rv0678* mutants showed a BDQ-MIC of 1 µg/mL. How these findings translate to the clinic is still to be determined. In particular, there are insufficient data to confirm that *Rv0678* mutations lead to treatment failure. In a recent study, 6/277 (2.2%) naïve patients had phenotypically resistant BDQ, of which 3 had mutations in *Rv0678*; however, sputum conversion was achieved in 5/6 patients^40^. In another study, MDR-TB patients with acquired BDQ resistance showing a mutation in *Rv0678* presented greater risk of treatment failure, although larger numbers are needed to confirm the correlation^41–43^. Also, BDQ has been shown to overcome baseline CFZ phenotypic resistance^43^. Urgent clinical studies are required to determine the true impact of low, moderate, and high phenotypic resistance to BDQ on treatment response. Additionally, similarly to HIV, differentiating primary mutations that confer resistance from secondary mutations that influence the fitness of the mutated strain could help guiding the individual patient management.

Amongst the *Rv0678* mutations identified in this study, 18 have been previously identified in DR-TB patients (both in exposed and naïve patients), confirming that in vitro experiments can (at least partly) select for clinically relevant mutations^44^. A recent study from South Africa showed that most BDQ-resistant patients presented a *Rv0678* mutation, 44% of which were concentrated in codon region 46 to 49, and codon 67^45^. Our study showed some mutations in the same region, but overall, our mutations were spread across the entire *Rv0678* gene.

Eighty-five out of 114 different *Rv0678* mutations described in this study have not been seen yet in clinical isolates or in vitro, possibly forecasting mutations that will be appearing in patients (suboptimally) treated with BDQ and/or CFZ. This seems to be particularly true for *atpE*, with all mutations described in this study corresponding to the ones reported in patients^12^. In fact, the BDQ bactericidal effect takes about one week to develop^46^; if accompanied by resistance to companion drugs, BDQ is insufficiently protected when the bacterial burden is highest, increasing the risk of acquired BDQ resistance. Also, BDQ has a long mean half-life of 5.5 months^47^. When patients interrupt treatment (risk factors for which include poverty, addiction, and experiencing major side effects), only BDQ remains in the serum, a condition that likely mimics the sub-lethal MIC mutant’s selection in vitro.

Interestingly, the synonymous Val1Val mutation in *Rv0678* gene was associated with elevated BDQ and CFZ MICs. This could be explained by the fact that only one of the four codons encoding for valine can act as a start codon (GCG)^48^. In our case, the nucleotide 3 G to A mutation would disrupt the start codon, prohibiting protein production. A similar G3A synonymous mutation abolishing the valine start codon has already been described for the *eis* gene, conferring resistance to amikacin and kanamycin^48^.

We describe 6 different mutations in the intergenic region between *Rv0678* and *mmpS5* (nucleotide position − 30, − 11, − 9, − 8), all associated with high BDQ and CFZ MICs. The transcriptional start site of Rv0678 was determined by 5’ RACE by Milano and colleagues, and it seems to be located directly upstream its translational start codon, suggesting that the -10 box could be located in the same region (TTTCAGAGTACAGTGAAA)^49^. Other studies performing DNA binding assays, suggest that the same region corresponds to the promoter DNA sequence^50^. Nevertheless, mutations in the intergenic region have been associated in the past with both decreased (position − 9, − 13) and increased (position − 11, − 44) susceptibility to BDQ^13,17^, confusing the interpretation of their impact on phenotypic BDQ/CFZ resistance.

In this study, we detected 4 novel mutations in the *pepQ* gene, which encodes for a putative Xaa-Pro aminopeptidase. Even if the function of this gene is still unknown, Alameda et al.^51^ suggested a mechanism through efflux. Mutations in *pepQ* have been reported to cause low cross resistance to BDQ and CFZ^38,51^, corroborated by our findings with *pepQ* mutations leading to increased MICs for both CFZ and BDQ generally above the critical concentration for CFZ but not for BDQ.

Finally, an important share (40,7%) of *Rv0678* mutations described in this study were indels; while frameshift mutations are generally expected to lead to non-functional, truncated proteins, in this study, frameshift mutations showed variable BDQ and CFZ MIC increase ranging from 4 to 32 fold. This may be explained by the fact that additional genes and mechanisms contribute to BDQ phenotypic resistance. However, another hypothesis is that frame-shifted protein could somehow retain the same structure and function as the wild type protein. Previous studies in other bacterial species have also observed similarities between frame-shifted and wild type proteins in term of structure and functionality^52^. It has been suggested that originally the coding genes could be translated into proteins using different reading frames, all leading to functional proteins^52^. While evolution would eventually select for the most efficient reading frame leading to the protein with best functionality, other reading frames would remain hidden, but available, helping the organism to tolerate frameshift mutations^53^. This could explain how some frameshift mutations in *Rv0678* reported in our study lead to limited MIC fold increase.

Despite showing the highest predicted protein instability, the *Rv0678_*C46R mutant is among the few *Rv0678* mutants that have been observed among patient isolates, potentially explicable by the fact that *Rv0678* is not an essential gene. The C46R mutation falls in the winged helix-turn-helix DNA binding domain, possibly affecting *Rv0678* functionality. On the other hand, all *atpE* mutations presented in this study were located in the BDQ binding pocket and showed low impact on protein stability. This may be related to the fact that *atpE* is an essential gene for Mtb, mutations impacting the protein stability would contribute to a too high fitness cost and are therefore not observed in cultured isolates.

Although protein stability seems to be somehow related to protein function, we could not find a general correlation between predicted protein instability and MIC increase for *Rv0678* mutations in this study. It is probable that other factors are contributing to function changes, for example whether or not the mutations occur in an active site of the protein^54^.

We acknowledge some limits in our study. First, the approach to selection of colonies adopted in this work did not allow robust estimation of mutation frequency. Secondly, the target sequencing approach only included *Rv0678*, *pepQ* and *atpE*, leaving out other candidate genes possibly associated with BDQ or CFZ resistance; due to time and cost restraints we performed additional Deeplex and WGS only on isolates presenting a WT *Rv0678/atpE* and *pepE* sequence. However, the great majority of isolates with high BDQ/CFZ MIC showed a mutation in one of these three genes, and our data confirm that mutations in *Rv0678* are the principal drivers of BDQ and CFZ resistance. Lastly, we acknowledge the significance of the transcriptional layer in evaluating the impact of *Rv0678* mutations on efflux pump expression and other genes. However, due to time constraints, our primary focus was directed towards the analysis of genomic and phenotypic data.

Current knowledge about which *Rv0678* mutations are related to resistance remains insufficient for DNA based diagnostic in clinical settings. Besides expanding BDQ/CFZ resistance-associated mutation datasets with increased attention to the contribution of promoter- and synonymous mutations in *Rv0678*, alternative approaches should be explored. Similarly to other bacteria, the phenotypic plasticity in Mtb could be mediated by changes in transcriptional profile^55^. Generation of additional layers of information (e.g., transcriptomics and proteomics data) are essential towards understanding the impact of *Rv0678* mutations on the MmpS5–MmpL5 efflux pump and deciphering regulatory mechanisms and yet unknown genes contributing to BDQ and CFZ drug resistance.

In conclusion, with our work we generate a broad catalogue of new mutations in *Rv0678* associated with phenotypic resistance, part of which have been deposited in the BCCM/ITM public collection^56^, and we provide insights on impact of mutations on protein stability. Our work brings us closer to bridging the gap in understanding the correlation between mutations and phenotypic BDQ resistance, which is crucial for development of molecular assays, treatment choice, and prevention of further emerging resistance to BDQ before its activity is lost. Future clinical studies should evaluate the real impact of low, moderate and high BDQ phenotypic resistance on treatment response.

## Supplementary Information

Supplementary Tables.

## Data Availability

The targeted DNA sequences generated during the current study are available in the ENA repository, PRJEB61455. Additional MIC and predicted protein stability data are available in the supplementary information.
